# Palliative pelvic exenteration using iliofemoral bypass with synthetic grafts for advanced cervical carcinoma

**DOI:** 10.4274/tjod.galenos.2018.66743

**Published:** 2019-03-27

**Authors:** Burak Tatar, Yakup Yalçın, Evrim Erdemoğlu

**Affiliations:** 1 University of Health Sciences, Samsun Training and Research Hospital, Clinic of Gynecologic Oncology, Samsun, Turkey; 2 Isparta State Hospital, Clinic of Gynecologic Oncology, Isparta, Turkey; 3 Suleyman Demirel University Faculty of Medicine, Department of Gynecologic Oncology, Isparta, Turkey

**Keywords:** Cervical cancer, iliofemoral bypass, palliation, pelvic exenteration

## Abstract

**Objective:**

Recurrent cervical cancer can cause severe morbidity. Despite the severe morbidity after surgery, pelvic exenteration is still used today for mainly curative intent. This intention is neither based on randomized controlled trials (RCTs) nor high quality non-RCTs with adequate patient numbers comparing medical management with surgery. The same is true for exenteration for palliative intent, so the patient selection for either curative or palliative intent must be considered on a patient-by-patient basis.

**Materials and Methods:**

A 35-year-old patient who had undergone primary chemo-radiotherapy for advanced cervical cancer presented with intractable pain on the swollen left leg and pelvis 8 months later. Left lower extremity Doppler ultrasound revealed echogenic thrombus in the external iliac, femoral, and popliteal veins, consistent with acute deep vein thrombus. She underwent total exenteration, end colostomy, ileal urinary conduit, pelvic lymphadenectomy, paraortic lymph node sampling, and ilio-femoral arterial and venous bypass.

**Results:**

The procedure relieved her pain, the leg diameter dramatically decreased from 75 cm to 44 cm, and circulation of the leg was reestablished. The procedure deferred leg amputation for about five months.

**Conclusion:**

To the best of our knowledge, this is the first report of a palliative pelvic exenteration for cervical cancer with combined iliofemoral arterial and venous bypasses. These procedures, with high morbidity and mortality, are also more controversial when undertaken for just palliation of symptoms. They must be considered in the basis of each patient, and the benefits and risks must be discussed thoroughly in a realistic perspective with the patient.

## Introduction

It is estimated that there are 527.600 new cervical cancer cases every year, nearly 265.700 deaths attributable to this malignancy, and most of the cases are seen in developing countries^(1)^. Most patients present at advanced stages and more than half of all patients with cervical cancer receive radiotherapy during the course of their treatment. Nearly one-third of patients who receive radiotherapy at any stage (stage I to stage IV) will have local or distant failure^(2)^. Recurrence after radiotherapy is maybe one of the most challenging situations in gynecologic oncology for patients with cervical cancer.

Recurrent or advanced cervical cancer can cause severe morbidity including intractable pain, continuous foul-smelling discharge, fecal and urinary incontinence due to fistula formation, vaginal bleeding, intestinal or ureteric obstruction related symptoms, and sepsis. In 1948, Brunshwig^(3)^ defined pelvic exenteration as, “a one-stage abdominoperineal operation with end colostomy and bilateral ureteral implantation into the colon above the colostomy,” to alleviate these symptoms for 22 patients. The perioperative mortality rate was 23%. Despite it having palliative intent when it was first defined, with the improvements in the surgical technique, especially with the use of modern urinary conduit technics, it has rather become a surgery for curative intent with much lower mortality rates^(4,5)^.

## Case Report

A 35-year-old patient previously underwent primary chemo-radiotherapy for a bulky [magnetic resonance imaging (MRI) revealed a mass of 70x65x35 mm] non-keratinizing squamous cell cervical carcinoma with invasion to the proximal one-third of the vagina and parametria, and a 50x30 mm lymph node chain, probably metastatic, on the left iliac chain according to MRI. She presented with intractable pain in the left leg and pelvis 8 months later.

**Figure 1:**
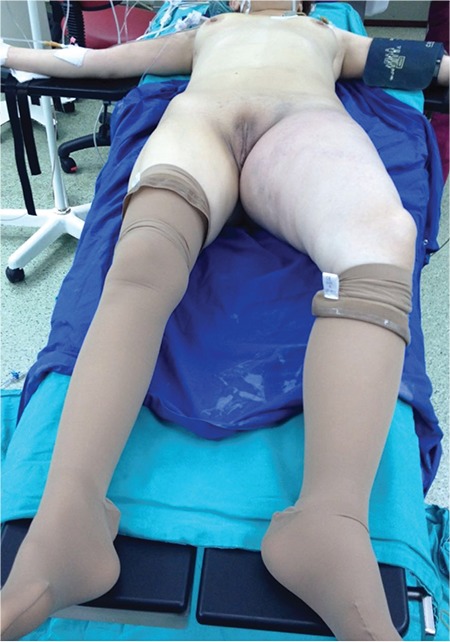
The image of the patient just before the procedure. Swollen left lower extremity is clearly seen

Her left leg was 75 cm in diameter, whereas its right counterpart was 40 cm at its maximum (Figure 1). Left lower extremity Doppler ultrasound revealed echogenic thrombus in the external iliac, femoral, and popliteal veins, consistent with acute deep vein thrombus.

**Figure 2:**
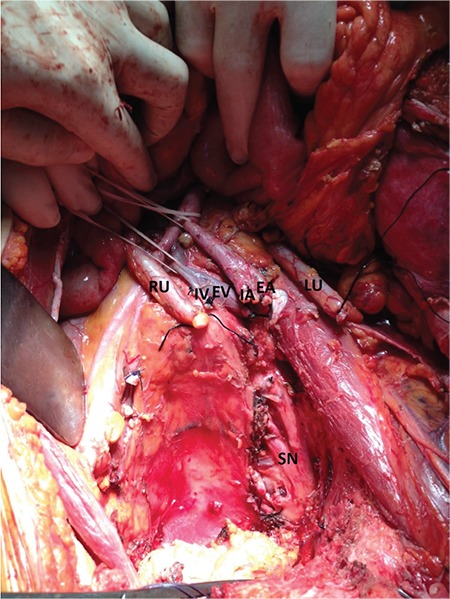
Before the reconstruction phase; operative field after total pelvic exenteration and left external iliac vessels ligated RU: Right ureter, IV: Internal iliac vein, EV: External iliac vein, IA: Internal iliac artery, EA: External iliac artery, LU: Left ureter, SN: Sacral nerve roots

**Figure 3:**
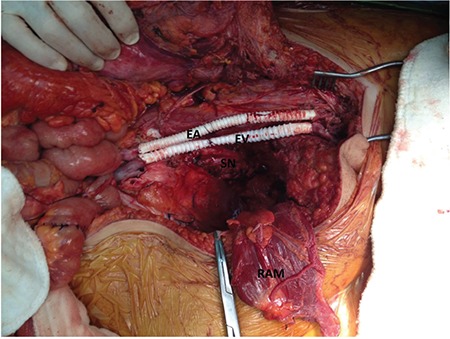
Operative field after iliofemoral arterial bypass and femoro-iliac venous bypass with polytetrafluoroethylene grafts EA: External iliac artery, EV: External iliac vein, SN: Sacral nerve roots, RAM: Rectus abdominis muscle before flap reconstruction

She was discussed in an oncology round, consulted by the cardiovascular surgery department, and the risks of the operation were discussed thoroughly, explaining no possible survival benefit, extreme risk of morbidity and mortality, and that the procedure would performed only for the possible alleviation of symptoms. She was fully cooperating and demanded the surgical intervention. The surgery was undertaken as total exenteration, end colostomy, ileal urinary conduit, pelvic lymphadenectomy, paraaortic lymph node sampling, and iliofemoral arterial and iliofemoral venous bypass. Intraoperatively, the tumor was visualized infiltrating the left external and internal iliac artery, left external and internal iliac vein, recto-sigmoid, bladder, and left ureter. The left ureter was seen as hydropic. In order to remove the tumor, the external and internal iliac artery, vein, and ureter were cut (Figure 2). With the help of an inguinal incision, the femoral artery and vein were identified, dissected, and cut. The backflow from the common femoral artery was confirmed and ilio-femoral arterial bypass was completed using an 8 mm ringed polytetrafluoroethylene (PTFE) graft. The thrombi in the femoral vein were cleared, and after the blood flow from the common femoral vein was confirmed, the femoro-iliac venous bypass was completed using a 10-mm PTFE graft (Figure 3). The flow from the distal part of the artery and back from the vein was confirmed. The right internal iliac artery was ligated and cut. The left presacral area was dissected and the sciatic nerve was preserved. The tumor was removed by stripping the pubis and ilium. After completing the exenteration and conduit, a prolene mesh was used to reconstruct the cut inguinal ligament.

The patient was discharged from hospital three weeks after the procedure, as her leg diameter dramatically decreased from 75 cm to 44 cm. The circulation of the leg was re-established and amputation was delayed until five months later when she was admitted to hospital for ischemic changes in her left foot and pain. The patient died of sepsis, approximately eight months after the palliative surgery.

## Discussion

After the initial description of pelvic exenteration by Brunshwig in 1948, there has been much debate about the surgery despite the refinement of the technique, especially for urinary conduits. The debate is about its indications, patient selection criteria, the technique, its aim, and its necessity. 

According to some articles after the 2000’s, survival after palliative pelvic exenteration is between 10.5% for 2 years to 27% for 5 years, whereas the reports from same authors indicate a 5-year survival between 50% and 60% if the exenteration is performed with curative intent^(4,6)^.

There is a more recent palliative pelvic exenteration series of 13 patients from Brazil, 9 of which were performed for recurrent cervical cancer. The 2-year overall survival was 15.4% and only 6 of 13 patients survived more than 5 months^(7)^.There is also a controversy about the definition of palliative pelvic exenteration. An early publication of Deckers et al.^ (8)^ defined pelvic exenteration as an efficient way to alleviate symptoms such as pain, fistulas, pelvic sepsis, hemorrhage, and malodorous discharge. Nevertheless, there is controversy about the definition of palliative pelvic exenteration. From the above-mentioned authors, Marnitz et al.^ (4)^ explained that the difference between palliative and curative exenterations could be discriminated by the resection margin status. Finlayson and Eisenberg emphasized three definitions of palliative exenteration in their review^(9)^. First, based on the intent that the objective is just for palliation of symptoms. Second, for patients who undergo surgery for curative intent but intraoperatively macroscopic tumor is left behind because of the non-resectability of the tumor. The third definition they found in the literature is that all surgical effort for failed primary curative effort including radiation, surgery or chemotherapy, which may be combined with each other. In the review of Hope and Pothuri^(10)^,  they mentioned that palliative exenteration surgery was accomplished to alleviate discomfort and not necessarily in an attempt to prolong life. They also stressed that the literature for palliative pelvic exenteration was not homogenous in the tumor, patient, and surgical intervention basis, making it difficult to compare. This problem was also documented in a recent Cochrane review for all exenteration procedures^(11)^.

In our case, because the tumor was in close proximity of the sacral plexus, the treatment was planned with palliative intent, not curative. Intraoperatively, the tumor was stripped from the nerve plexus. This region is the boundary between curative intent of laterally extended endopelvic resection as described by Höckel^(12)^ and palliative surgery.

There is a controversy over all kinds of pelvic exenterations for gynecologic malignancies; their indications are not clear, the surgical procedures are not uniform, and most importantly, their efficacy over non-surgical treatments are not proven. Chemotherapy may be an alternative for palliation of symptoms to surgery, but there are no randomized controlled trials comparing one with the other in the literature. Full recovery, if possible, from a palliative exenteration may take about 4-5 months, survival may not be much more and quality of life during this period is poor.

There is a case series from Memorial Sloan Kettering Cancer Center; 11 patients with recurrent uterine cancer and 3 with recurrent cervical cancer underwent pelvic exenteration for curative intent. Two of the patients had femoral-femoral arterial bypass procedures. The specific survival and prognosis of these two patients is not mentioned^(13)^.

A recent report from Romania described palliative posterior pelvic exenteration with partial cystectomy for a tumor invading the sciatic foramen for fistula after a previous radical hysterectomy^(14)^. No detail was included regarding the prognosis or survival of the patient in the article.

To the best of our knowledge, this is the first report of a palliative pelvic exenteration for cervical cancer with combined iliofemoral arterial and venous bypasses.

Under these circumstances, such procedures with high morbidity and mortality are also more controversial when undertaken simply for palliation of symptoms. They must be considered on a patient-by-patient basis, and the benefits and risks must be discussed thoroughly in a realistic perspective, taking into account the physical and emotional aspects of the patient before planning the procedure.
